# Regulation of Exocyst Function in Pollen Tube Growth by Phosphorylation of Exocyst Subunit EXO70C2

**DOI:** 10.3389/fpls.2020.609600

**Published:** 2021-01-14

**Authors:** Antonietta Saccomanno, Martin Potocký, Přemysl Pejchar, Michal Hála, Hiromasa Shikata, Claus Schwechheimer, Viktor Žárský

**Affiliations:** ^1^Laboratory of Cell Biology, Institute of Experimental Botany, Czech Academy of Sciences, Prague, Czechia; ^2^Department of Experimental Plant Biology, Faculty of Science, Charles University, Prague, Czechia; ^3^Plant Systems Biology, Technische Universität München, Freising, Germany

**Keywords:** exocyst, phosphorylation, **p**ollen tube, membrane trafficking, secretion inhibitor, tip-growth, Exo70

## Abstract

Exocyst is a heterooctameric protein complex crucial for the tethering of secretory vesicles to the plasma membrane during exocytosis. Compared to other eukaryotes, exocyst subunit EXO70 is represented by many isoforms in land plants whose cell biological and biological roles, as well as modes of regulation remain largely unknown. Here, we present data on the phospho-regulation of exocyst isoform EXO70C2, which we previously identified as a putative negative regulator of exocyst function in pollen tube growth. A comprehensive phosphoproteomic analysis revealed phosphorylation of EXO70C2 at multiple sites. We have now performed localization and functional studies of phospho-dead and phospho-mimetic variants of Arabidopsis EXO70C2 in transiently transformed tobacco pollen tubes and stably transformed Arabidopsis wild type and *exo70C2* mutant plants. Our data reveal a dose-dependent effect of At*EXO70C2* overexpression on pollen tube growth rate and cellular architecture. We show that changes of the AtEXO70C2 phosphorylation status lead to distinct outcomes in wild type and *exo70c2* mutant cells, suggesting a complex regulatory pattern. On the other side, phosphorylation does not affect the cytoplasmic localization of AtEXO70C2 or its interaction with putative secretion inhibitor ROH1 in the yeast two-hybrid system.

## Introduction

The heterooctameric protein complex exocyst is a major component of exocytosis in eukaryotes composed of eight subunits: Sec3, Sec5, Sec6, Sec8, Sec10, Sec15, Exo70, and Exo84. Exocyst mediates the first contact and subsequent tethering of post Golgi vesicles with the plasma membrane, and, through interactions with SNARE proteins, it facilitates the final fusion of vesicles with the plasma membrane (Heider and Munson, 2012; Mei and Guo, 2018; Rossi et al., 2020). Exocyst-mediated vesicle targeting is crucial in plant cell morphogenesis as well as in the biogenesis of interfaces among plant cells and for interactions between the plant and its environment (Zárský et al., 2009, 2013).

Exocyst was originally identified in *Saccharomyces cerevisiae* by biochemical and genetic approaches as an interactor of the activated Rab GTPase Sec4 (TerBush et al., 1996). Exocyst is conserved also in animals and plants (Hsu et al., 1996; Hála et al., 2008), and its origin can be traced back to a common eukaryotic ancestor (Koumandou et al., 2007; Heider and Munson, 2012; Vaškovičová et al., 2013; Vukašinović and Žárský, 2016). Studies in yeast, mammalian and plant cells revealed that SEC3 and EXO70 subunits localize exocyst to specific plasma membrane domains through interactions with phosphoinositides, specifically with phosphatidylinositol 4,5-bisphosphate (He et al., 2007; Liu et al., 2007; Wu et al., 2010; Pleskot et al., 2015; Bloch et al., 2016) and that exocyst-phospholipid binding contributes to a successful establishment and maintenance of cell polarity also in plant cells (Sekereš et al., 2017; Kubátová et al., 2019).

*In silico* analyses revealed 23 genes encoding EXO70 isoforms in *Arabidopsis thaliana* and even 47 in rice, suggesting a functional diversification of EXO70 function in higher plants (Synek et al., 2006; Cvrcková et al., 2012). The EXO70 paralogs can be subdivided into three well-separated ancient monophyletic clades: EXO70.1, EXO70.2, and EXO70.3 (Synek et al., 2006; Chong et al., 2010; Zhang et al., 2010; Cvrcková et al., 2012; Žárský et al., 2020), a diversification that is already apparent in the bryophyte *Marchantia polymorpha*, where each clade is represented just by one paralog (Rawat et al., 2017). Specifically for clade EXO70.2, diversification seems to be driven by the evolutionary arms race between plants and their pathogens (Žárský et al., 2020).

Pollen tubes, as well as root hairs, are well accepted model systems to study exocytosis, which is regulated by ROP GTPases and membrane phospholipid-modifying enzymes (Vaškovičová et al., 2013; Qin and Dong, 2015). During polar (tip) growth, pollen tubes rapidly elongate to deliver two sperm cells to the female gametophyte for fertilization. Seven *EXO70* isoforms are expressed in Arabidopsis and tobacco pollen, and *EXO70C* paralogs were the most abundant exocyst subunit-encoding transcripts in transcriptomics studies on pollen from both species (Lai, 2016; Sekereš et al., 2017). *EXO70C* paralogs are also highly expressed in root hairs (trichoblasts), indicating a possible common role of EXO70C in polar tip growth in pollen and root hairs (Synek et al., 2017).

Disrupted polarization and cell growth in different plant cell types were observed in exocyst mutant plants in evolutionary distinct plant species and several exocyst isoforms are essential for pollen tube growth (Hála et al., 2008; Sekereš et al., 2017; Synek et al., 2017). Arabidopsis loss-of-function (LOF) mutants of *SEC3a*, *SEC5a/b*, *SEC6*, *SEC8*, or *SEC15a* have very short and depolarized pollen tubes, which correlates with a failure to transmit the mutant alleles through the male gamete (Cole et al., 2005; Hála et al., 2008; Bloch et al., 2016). The two *EXO70A* isoforms, *EXO70A1* (Synek et al., 2006) and *EXO70A2* (Marković et al., 2020), seem to have a general housekeeping function during exocytosis in the sporophyte and the male gametophyte, respectively. Partially resembling the phenotypes of LOF mutants of core exocyst subunits, *exo70A2* pollen grains germinate poorly and produce short, thick, slowly growing pollen tubes (Beuder et al., 2020; Marković et al., 2020). In sharp contrast, LOF of Arabidopsis *EXO70C2* results in stop-and-go growth dynamics where phases with abnormally high growth rates are interrupted by pollen tube tip bursts and recovery, ultimately resulting in pollen tube rupture (Synek et al., 2017). EXO70C1 or EXO70C2 do not interact with the core exocyst subunits in the yeast two-hybrid assay, are localized predominantly in the cytoplasm, and genetic analysis of LOF mutants in Arabidopsis, as well as over-expression experiments in tobacco, suggest that these unconventional exocyst subunits have negative regulatory function during pollen tube elongation (Sekereš et al., 2017; Synek et al., 2017). Moreover, the EXO70A1, EXO70C1, and EXO70C2 exocyst subunits interact with ROH1, a putative negative regulator of secretion, in the yeast two-hybrid assay (Kulich et al., 2010).

Protein phosphorylation contributes significantly to the regulation of exocyst function in yeast and animals (Chen et al., 2011; Luo et al., 2013; Lepore et al., 2016). In mammals, Sec5 phosphorylation participates in exocyst-dependent GLUT4 recycling (Chen et al., 2011), insulin stimulates Sec8 phosphorylation (Lyons et al., 2009) and Sec8 phosphorylation participates in exocyst recruitment to neurite growth cones (Chernyshova et al., 2011). Phosphorylation of mammalian Exo70 substantially stimulates exocyst assembly and exocytosis (Ren and Guo, 2012). Exo84 phosphorylation by TBK1 kinase promotes insulin-stimulated GLUT4 exocytosis (Uhm et al., 2017), and in the fungus *Candida albicans* Cdk1-Hgc1-mediated phosphorylation of Exo84 contributes to hyphal extension – a process analogous to pollen tube or root hair tip growth (Caballero-Lima and Sudbery, 2014). Relevant is also the inhibitory phosphorylation of the yeast exocyst subunit Exo84 by CDKs, disrupting exocyst assembly and resulting in growth arrest (Luo et al., 2013).

While no study focusing on exocyst subunit phosphorylation in plants has been reported to date, an extensive Arabidopsis phosphoproteome analysis revealed SEC5 and SEC10 exocyst subunits as phosphorylation substrates, surprisingly in purified nuclei (Jones et al., 2009). Further evidence for exocyst subunit phosphorylation, including phosphorylation of EXO70C2, came from a comprehensive proteome and phosphoproteome analysis conducted in *Arabidopsis thaliana* as discussed below (Mergner et al., 2020).

Here, we examine the role of EXO70C2 for exocyst function in tobacco pollen tubes using antisense oligodeoxynucleotides (AODNs) against *EXO70C2* and reveal a negative function for *EXO70C2* in pollen tube growth as is the case in Arabidopsis (Synek et al., 2017). We then examined the functional significance of EXO70C2 phosphorylation for its inhibitory action in pollen tube growth by performing comparative analyses of the effect of wild type, phospho-dead (PD) and phospho-mimetic (PM) variants of EXO70C2 on the tip growth in tobacco and Arabidopsis pollen tubes. Our results suggest that EXO70C2 phosphorylation regulates its inhibitory function during pollen tube growth and elongation.

## Materials and Methods

### Design of Antisense Oligodeoxynucleotides and Pollen Tube Treatment

Oligodeoxynucleotides (ODNs) were designed from tobacco *EXO70C2* sequences (Sekereš et al., 2017) to generate specific and functional heteroduplexes with RNA to avoid possible secondary structure formations. The Soligo software program^1^ was used to predict secondary structures of antisense oligodeoxynucleotides (AODNs). The two best scoring AODNs, AS1 (5′-TTGTTGGGATCATCTTCTTG-3′) and AS2 (5′-CCCAACTGCTTTGTGTTTTA-3′), and the corresponding sense control ODNs, S1 (5′-CAAGAAGATGATCCCAACAA-3′) and S2 (5′-TAAAACACAAAGCAGTTGGG-3′) were synthetized with phosphorothioate modifications at the 5′- and 3- termini. Selectivity of AODNs for the target gene was verified by BLAST^2^. Lyophilized ODNs were rehydrated with sterile double-distilled water to obtain 1 mM stocks and stored at −20°C.

Tobacco pollen grains supplemented with the various ODNs (50 μM final concentration) were grown *in vitro* in minimal PEG liquid media (20% polyethyleneglycol 3350, 1.6 mM H_3_BO_3_) for 3 h (Potocký et al., 2019). Pollen tubes were examined with a Zeiss Axioimager (HPX120V excitation, with camera Axiocam 506 mono) with Achromat 5 dry objective and microscope settings were kept constant to allow comparative analysis between the images and between the replicates.

### Molecular Cloning

Arabidopsis (*Arabidopsis thaliana*) *EXO70C2* (AT5G13990) CDS was amplified from the construct pEXO70C2: EXO70C2:GFP (Synek et al., 2017). The construct was first cloned into the pJET vector and then transferred to the final vectors. Variants of the protein were generated replacing threonines (212, 446) and serines (215, 217, 494, 605) by alanines for the AtEXO70C2 phospho-dead (PD) and, by glutamic acid and aspartic acid, respectively, for the At*EXO70C2* phospho-mimetic (PM) variant. All coding sequences were PCR-amplified using Q5 High-Fidelity DNA Polymerase (NEB) and flanked by *Ngo*MIV and *Apa*I. To obtain an N-terminal YFP fusion, the *EXO70C2* CDS was inserted into corresponding cloning sites of the pollen-specific expression vector pWEN240 (LAT52:YFP-GA5-MCS:NOS) and to obtain a C-terminal YFP fusion into pHD32 (LAT52:MCS-GA5-YFP:NOS) (Klahre et al., 2006). To obtain Arabidopsis plants expressing pEXO70C2:EXO70C2-PD:GFP and pEXO70C2:EXO70C2-PM:GFP, the corresponding part of coding sequence in the wild type pENTR3C Gateway construct (Synek et al., 2017) was replaced by *Kpn*I/*Eco*RV fragments from EXO70C2-PD and -PM variants in the pJET vector. Sequences were further transferred using LR Clonase II (Invitrogen) to the pB7FWG0 Gateway vector and the final binary constructs were then transformed by *A. tumefaciens*-mediated transformation to Arabidopsis Col-0 wild type and *exo70C2* homozygous mutant plants.

For yeast-two hybrid analysis, coding sequences of *ROH1A* (AT1G63930) were amplified from *Arabidopsis thaliana* Col-0 genomic DNA using primers 5′-TCTTGTACAAAGTGGAACAT ATGAGACCTGCGCAAGAT-3′ and 5′-TGTATAATAAAGTTG GATCCTTACACAACTGGCGCCG-3′, and *ROH1D* (AT1G- 74450) using primers 5′-TCTTGTACAAAGTGGAATTCTTGA GGATGCCAGCAAC-3′ and 5′-TGTATAATAAAGTTGGTCGA CTCATTCAGAACCATGATG-3′. The inserts were fused with the GAL4 DNA-binding domain in the pGBKT7 vector. Cloning of AtEXO70C2-PD and -PM variants fused with the GAL4 activation domain in pGADT7 was done as described in Synek et al. (2017), using gene fragments as described above.

### Transient Pollen Transformation and Microscopic Analysis

Pollen was collected from outdoor- or glasshouse-grown *Nicotiana tabacum* cv. *Samsun* flowers before opening of the flowers during warm and dry weather and kept frozen at −20°C until further use. For each biolistic pollen transformation, 1 mg of pollen grains germinating on solid pollen culture medium was used and bombardment with DNA-coated gold was performed using a particle delivery system (PDS-1000/He; Bio-Rad) at 1100 psi (Kost et al., 1998). For subcellular protein localization studies, 2–6 μg of plasmid DNA was used and 5–8 h-old germinated pollen tubes were analyzed with a spinning disk confocal microscope (Yokogawa CSU-X1 on Nikon Ti-E platform, laser box Agilent MLC400 with sCMOS camera PRIM 95B Photometrics) using a Plan Apochromat 40x WI objective and a 488 nm laser line. Time lapse series images were taken for 1 min at 2 s intervals. Camera and microscope settings were kept constant to allow for comparative imaging.

### Quantification

Quantification of tobacco and Arabidopsis pollen tube lengths was carried out by measuring the distances from the pollen grain to the pollen tube tip using segmented line and length measurements with the ImageJ software (Schindelin et al., 2015).

Measurements of pollen tube growth rate and thickness were conducted manually on at least 50 transformed tobacco pollen tube per variant from three independent experiments using segmented line and length measurement tools of the ImageJ software. For the evaluation of the YFP-tagged intensity signal for the transgenic proteins, background signal was subtracted and maximum intensity values of individual images obtained with the same acquisition settings were measured after an arbitrary threshold had been empirically set for the signal intensity spectrum. For the categorization of pollen tubes with typical and atypical morphological phenotypes, different pollen tub tip-shape categories were determined.

Data are presented as box plots prepared using the ggplot2 package in R^3^ where the horizontal line in each box represents the median, the top and the bottom lines of the box including 75th and 25th percentiles, and the higher and lower lines represent extreme values. Experimental values were analyzed with the agricolae package in R using Kruskal–Wallis or Dunn’s *post hoc* tests with the Benjamini–Hochberg correction to test for significant differences at *P* < 0.05.

### Bioinformatic Analysis of Phosphorylation Motifs and Structural Homology Modeling

To analyze the evolutionary conservation of phosphosites in plant EXO70s, a sequence set from our previous study (Rawat et al., 2017) was updated and EXO70 orthologs from 16 additional angiosperm genomes were included. These new sequences were obtained by BLAST searches of the Phytozome v12 database (Goodstein et al., 2012) using Arabidopsis EXO70C1 and EXO70C2 protein sequences as queries. The full list of sequences is provided in Supplementary Figure S1. Protein sequence alignment was performed using the MAFFT G-INS-I algorithm (Katoh and Standley, 2013) in the Jalview software (Waterhouse et al., 2009) and maximum-likelihood phylogeny reconstructed as described in Marković et al. (2020). Final sequence conservation analysis of the phosphosites was performed on 23 EXO70C1 and 20 EXO70C2 paralogs and orthologs from 17 dicot genomes using WebLogo algorithm (Crooks et al., 2004). EXO70C2 orthologs from the following organisms were used for the final WebLogo analysis: *Arabidopsis thaliana, Arabidopsis lyrata, Brassica rapa, Capsella grandiflora, Carica papaya, Daucus carota, Eucalyptus grandis, Medicago truncatula, Nicotiana tabacum, Phaseolus vulgaris, Populus trichocarpa, Prunus persica, Ricinus communis, Solanum lycopersicum*, and *Theobroma cacao*.

The homology model for EXO70C2 was built independently using Modeller 9v17 and Robetta algorithms (Kim et al., 2004; Webb and Sali, 2016). The best models from both methods were very similar with backbone root-mean-square-deviation (RMSD) values < 2 Å. Robetta models were used for further analyses. Models of phospho-dead (PD) and phospho-mimetic (PM) EXO70C2 variants were calculated in Modeller using a custom-made Python script. Images were prepared using Pymol and Inkscape software packages (Yuan et al., 2016).

### *In vitro* Arabidopsis Pollen Germination and Imaging

Pollen was germinated on Lab-Tek II microscopic coverslides (Thermo Scientific) coated with thin layer of germination medium [10% sucrose, 5 mM KCl, 0.01% (w/v) H_3_BO_3_, 5 mM CaCl_2_, 1 mM MgSO_4_, and 1 mM Ca(NO_3_)_2_, pH adjusted to 7.5] solidified with 1.5% low-melting agarose (Duchefa). Pollen grains from 2 to 3 fully opened flowers were spread onto each slide. Slides were enclosed in a humid chamber and incubated in a plant growth room at 22°C for 16 h (Boavida and McCormick, 2007). Pollen tubes were examined with a Zeiss Axioimager (HPX120V excitation, with an Axiocam 506 mono camera) with an Achromat 10 dry objective. Microscope settings were kept constant to allow comparative analyses between the images and between the replicates.

### Yeast Two-Hybrid Assay

Yeast two-hybrid screening was carried out using the MATCHMAKER GAL4 Two-Hybrid System (Clontech) with all procedures following manufacturer protocols. Yeast strain AH109 (*MATa*, *trp1-109, leu2-3, 112, ura3-52, his3-200, gal4*Δ, *gal80*Δ, *LYS2:GAL1_*UAS*_-GAL1_*TATA*_-HIS3, MEL1, GAL2_*UAS*_-GAL2_*TATA*_-ADE2, URA3:MEL1_*UAS*_-MEL1_*TATA*_-lacZ*) was transformed first with BD:ROH1A or BD:ROH1D and then with AD:AtEXO70C2 wild type or its phospho-mimetic (PM) or phospho-dead (PD) variants. Transformed yeast single colonies were diluted in sterile water to obtain OD_600_ = 2 and serial dilutions were made by repeated 30x dilutions. From each dilution, a 12 μl droplet was applied to plates containing -Trp-Leu, -Trp-Leu-His, or -Trp-Leu-His-Ade drop-out growth media and plates were incubated in 30°C for 3 days before scoring.

## Results

### EXO70C2 Knock-Down by Antisense Oligodeoxynucleotides Stimulates Tobacco Pollen Tube Growth *in vitro*

**FIGURE 1:**
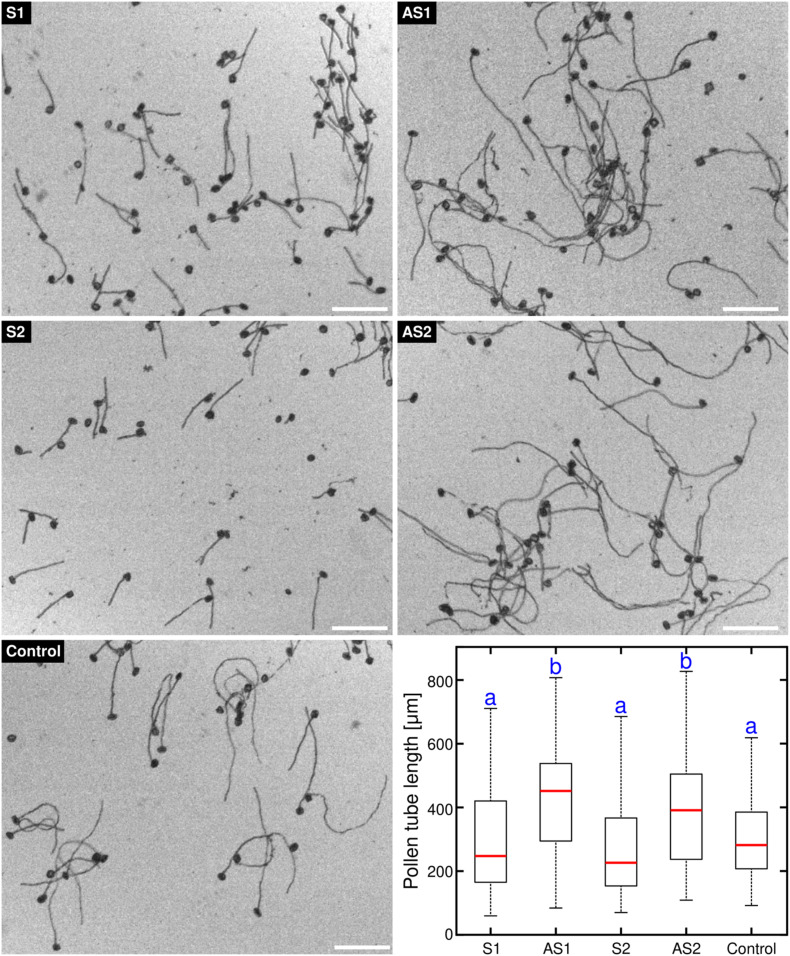
Antisense oligonucleotide-mediated knock-down of tobacco *EXO70C2* leads to faster pollen tube growth. Tobacco pollen tubes were incubated with 50 μM of two different antisense **(AS1,AS2)** and corresponding sense **(S1,S2)** oligodeoxynucleotides specific for *EXO70C2*. Non-treated wild type pollen was used as control. Microscopic images and boxplot show data from a representative experiment that was repeated twice with identical trend. At least 140 cells were measured per treatment. Different letters indicate significant differences between samples (*P* < 0.001). Bar, 200 μm.

We had previously observed that the pollen tube growth rate was enhanced in an *Arabidopsis thaliana exo70C2* mutant line (Synek et al., 2017). In order to test whether the role of *EXO70C2* in pollen is conserved in tobacco, we suppressed the endogenous *EXO70C2* gene function in germinating tobacco pollen by transformation with antisense oligodeoxynucleotides (AODNs), which leads to heteroduplex formation with the *EXO70C2* target mRNA and subsequent degradation through cleavage by RNAse H (Sun et al., 2005). Our data showed that, in contrast to control and sense ODNs-treated cells, the treatment of germinating tobacco pollen with both EXO70C2-directed AODNs distinctly stimulated pollen tube growth, suggesting that *EXO70C2* is a negative regulator of pollen tube elongation in tobacco (Figure 1). This suggests that the role of *EXO70C2* is conserved between Arabidopsis and tobacco (Synek et al., 2017).

### Phosphoproteomic and *in silico* Analyses Suggest Different Evolution of Phosphosites in EXO70C2 Subfamily in Dicots

**FIGURE 2:**
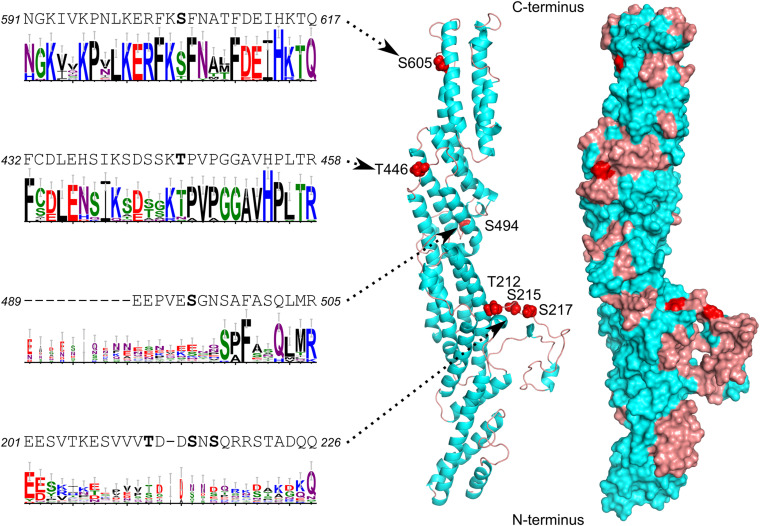
Homology model of Arabidopsis *EXO70C2* showing the positions of studied phosphosites, their adjacent sequences and sequence logos illustrating the evolutionary conservation within dicots. The structure is shown as ribbon and surface representation with alpha-helices in cyan, loops in brown and phosphosites in red. See Supplementary Figure S1 for the full alignment and additional details.

To examine the role of EXO70C subunits in pollen development, we searched available omics data for the expression of *EXO70C* isoforms. Our analysis of transcriptomic (Zimmermann et al., 2004; Winter et al., 2007; Loraine et al., 2013), as well as proteome data (Grobei et al., 2009; Mergner et al., 2020) revealed that *EXO70C1* and *EXO70C2* are the most abundant EXO70 isoforms in pollen. Particularly, *EXO70C2* was strongly expressed at later stages of pollen development when examined by real-time PCR (Lai, 2016; Synek et al., 2017). The strong abundance of both *EXO70C1* and *EXO70C2* had also been detected in tobacco pollen (Sekereš et al., 2017). Analysis of available pollen phosphoproteomes (Mayank et al., 2012; Mergner et al., 2020) further showed that EXO70C2 subunits are subject to phosphorylation at amino acid positions Threonine (T) 212, Serine (S) 215, S217, T446, S494, and S605 (Figure 2). To gain insight into the possible functional implications of EXO70C2 phosphorylation, we mapped the identified phosphosites onto the 3D homology model of near full-length AtEXO70C2 (amino acids 120–682). All six phosphosites are located on the surface of the modeled protein structure (Figure 2) and *in silico* mutation of all phosphosites to either alanine (phospho-dead mutation, PD) or aspartate/glutamate (phospho-mimetic mutation, PM) showed no major effect on the overall protein model or surface charge distribution (data not shown). When we analyzed the spatial distribution of the phosphosites on EXO70C2 surface, we observed that the phosphosites were scattered around the surface and most of them, five out of six, were located in the loop or linker regions with no secondary structure (Figure 2).

To unravel the conservation of the six phosphosites and their adjacent sequences in plant EXO70s, and especially within the C1 and C2 subfamilies, we analyzed 43 EXO70C sequences from 19 genomes that span the spectrum of dicot diversity. The data, presented as sequence logos that illustrate the level of conservation, clearly show that four of the phosphosites, T212, S215, S217, and S494, are located in the non-conserved regions of the protein and are not present in EXO70Cs from other *Brassicaceae* species (Figure 2 and Supplementary Figure S1). On the other hand, T446 is part of the very conserved region and is retained not only in most EXO70C1 and EXO70C2 homologs but also in many other EXO70 subfamilies. Notably, S605 is also located within a region that is conserved across EXO70s but the phosphosite can only be found in EXO70C2 orthologs and is not present in the EXO70C1 sister group (Figure 2 and Supplementary Figure S1). Taken together, our bioinformatic analysis suggests that phosphorylation of EXO70C2 occurs at conserved and isoform-specific sites that are distributed along the surface of the EXO70C2 protein.

### A Free EXO70C2 C-terminus Is Important for Its Negative Regulatory Effect on Pollen Tube Growth and Polarity

**FIGURE 3:**
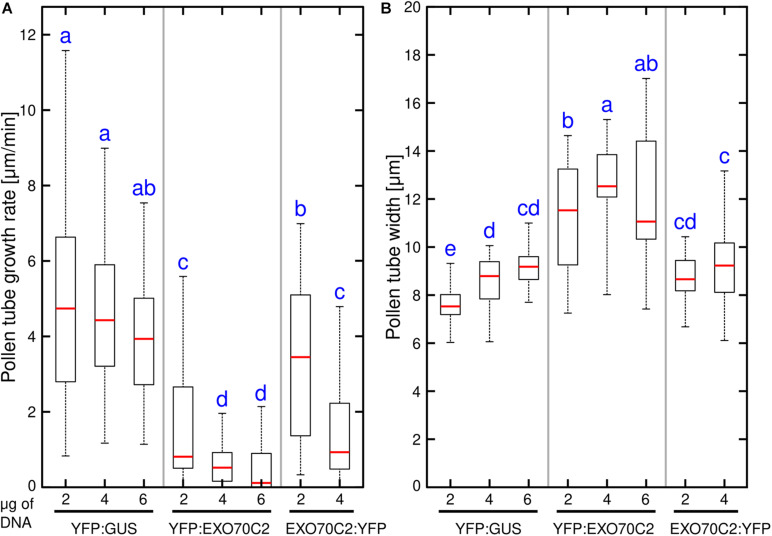
Expression of Arabidopsis EXO70C2 in tobacco pollen tubes alters pollen tube growth rate **(A)** and thickness **(B)** in dose-dependent manner. Three different DNA amounts (2, 4, and 6 μg) of constructs coding for wild type YFP:AtEXO70C2 or AtEXO70C2:YFP were transiently expressed in *Nicotiana tabacum* pollen tubes. Pollen tubes expressing YFP:GUS were used as a control. Measurements were performed on at least 40 transformed growing cells per each construct imaged in three independent experiments. Different letters indicate significant differences between samples at *P* < 0.05.

We have previously described that the overexpression of tobacco *EXO70C2* in tobacco pollen tubes leads to growth arrest and pollen tube defects (Sekereš et al., 2017). Here, we tested whether the same effect can be observed after expression of the *Arabidopsis thaliana* EXO70C2 ortholog in tobacco pollen. To this end, we generated N-terminal and C- terminal fusions with yellow fluorescent protein (YFP) of the Arabidopsis EXO70C2 isoform and expressed the fusion proteins from the pollen-specific *LAT52* promoter. Pollen growth rate and width were measured as simple proxy parameters for the efficiency and spatial regulation of exocytosis, respectively. Observations were conducted using spinning disk confocal microscopy and YFP:GUS expressed from the same promoter was used as control. In agreement with our previous studies (Sekereš et al., 2017; Synek et al., 2017), both N-terminal and C-terminal EXO70C2 fusions localized to the cytoplasm in pollen tubes. To test the dosage effect of the fusion proteins on tobacco pollen tube growth and width, we transformed tobacco pollen with three different DNA concentrations (2, 4, and 6 μg) of the respective plasmids. Overexpression of both, N- and C- terminal, YFP fusions of EXO70C2 led to a significant, dose-dependent reduction of the pollen tube growth rates when compared to the YFP:GUS control. These experiments showed much stronger effect on tip growth when EXO70C2 was tagged at the N-terminus than when tagged at the C-terminus (Figure 3A). The latter observation correlated with higher fluorescence signal intensity of the N-terminal fusion protein, suggesting reduced stability of the EXO70C2 C-terminal fusion.

The measurements of the pollen tube width in the subapical region revealed a similar trend as observed for growth rate measurements, as we noticed an increased pollen tube width correlating with pollen tube growth inhibition when we transformed cells with higher *EXO70C2* amounts (Figure 3B). This indicates a dose-dependent negative effect of EXO70C2 on cell expansion. Although EXO70C2:YFP exhibited a similar inhibitory trend as YFP:EXO70C2 for both parameters on tobacco pollen tube growth, the final effect of the overexpression of *EXO70C2:YFP* was mild when compared to the overexpression of *YFP:EXO70C2*. This might indicate that EXO70C2 tagged at the C-terminus is functional, but that C-terminal tagging probably interferes with the normal activity of the protein or with protein homeostasis.

### Overexpression of an AtEXO70C2 Phospho-Dead Variant Alleviates Its Inhibitory Effects on Pollen Tube Growth

To study the function and the physiological relevance of the phosphorylation of EXO70C2, we took advantage of the fact, that several phosphosites found experimentally in Arabidopsis EXO70C2 are conserved also in tobacco and we performed analysis of tobacco pollen tube growth and subcellular localization after transient expression of wild type (YFP:AtEXO70C2-WT), phospho-dead (YFP:AtEXO70C2-PD) and phospho-mimetic (YFP:AtEXO70C2-PM) AtEXO70C2 variants. In YFP:AtEXO70C2-PD and -PM variants, all EXO70C2 phoshorylated amino acids residues, namely Ser 215, 217, 494, 605 and Thr 212, 446, were substituted either by Ala (PD) or by Asp/Glu (PM). YFP:GUS and pollen-specific AtEXO70A2 were used as controls (Sekereš et al., 2017; Synek et al., 2017).

**FIGURE 4:**
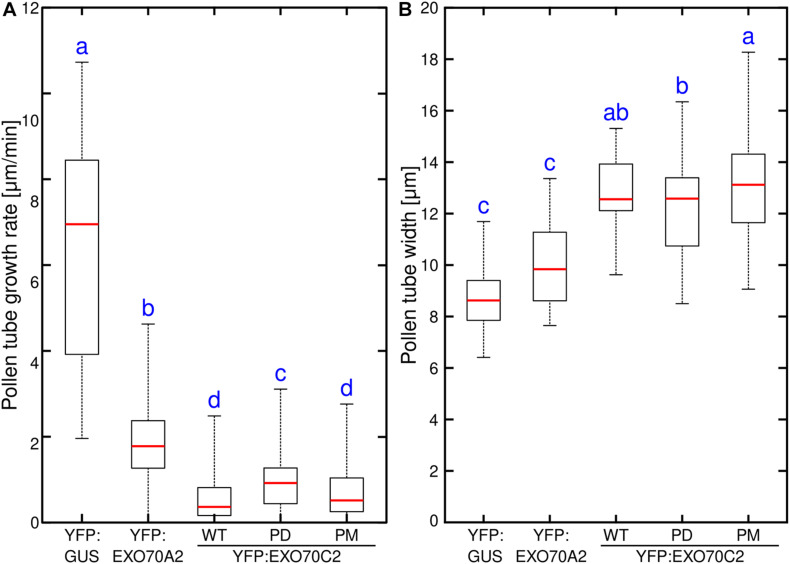
Phosphorylation of EXO70C2 regulates its inhibitory function in tobacco pollen tubes. Quantitative analysis of growth rate **(A)** and thickness **(B)** in tobacco pollen transiently expressing 4 μg of YFP:AtEXO70C2 wild type (WT), phospho-dead (PD) and phospho-mimetic (PM) variants. Pollen tubes expressing YFP:GUS and Arabidopsis YFP:EXO70A2 were used for comparison. Measurements were performed on 40 or more transformed pollen tubes per variant imaged during three independent experiments. Different letters indicate significant differences between samples at *P* < 0.05.

We observed that expression of the *EXO70C2-PM* variant inhibited pollen tube growth to the same extent as *EXO70C2-WT* (Figure 4A). On the other hand, expression of the *EXO70C2-PD* version showed distinctly less efficient inhibition compared to the other two variants (Figure 4A). Strong overexpression of all three *EXO70C2* variants generated pronounced abnormal pollen tube growth that correlated with increased pollen tube thickness, indicating a dose-dependent inhibitory effect (Figure 4). In contrast to previous observations, also expression of *AtEXO70A2* had an inhibitory effect in these assays, but with a distinctly lower inhibitory capacity than observed for the *AtEXO70C2* variants.

**FIGURE 5:**
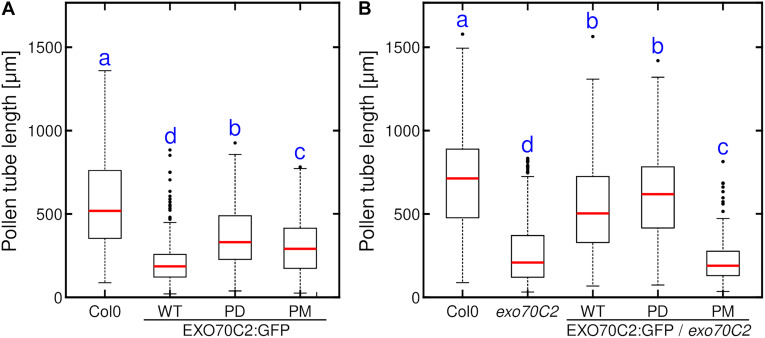
Expression of phospho-dead EXO70C2 differently affects the growth of Arabidopsis wild type and *exo70C2* mutant pollen tubes. Quantitative analysis of pollen tube length of Arabidopsis wild type (WT) **(A)** and *exo70C2* mutant **(B)** expressing EXO70C2p:AtEXO70C2:GFP wild type, phospho-dead (PD), and phospho-mimetic (PM) variants. Pollen tubes from Col-0 ecotype and *exo70C2* mutant that did not express any GFP-fused AtEXO70C2 variant were used for comparison as positive and negative controls, respectively. Measurements were performed on 148 or more pollen tubes for each variant imaged during three independent experiments. Different letters indicate significant differences between samples at *P* < 0.05.

Having established that the changes in AtEXO70C2 phosphorylation status can differently modulate the growth of tobacco pollen tubes, we tested their effect in stably transformed Arabidopsis wild type plants. We predicted that, similarly to the situation seen in tobacco, there would be a gene dosage effect in Arabidopsis pollen. We therefore chose the native *EXO70C2* promoter instead of the strong *LAT52* promoter to get only mild overexpression outcomes. Analysis of pollen tube lengths of Arabidopsis pollen tubes grown *in vitro* for 16 h showed that in agreement with the tobacco data, expression of *EXO70C2-WT* or *EXO70C2-PM* in Col-0 plants led to pronounced pollen tube growth inhibition while the overexpression of *EXO70C2-PD* variant had a significantly milder effect (Figure 5A).

### The Pollen Tube Length Defect of the Arabidopsis *exo70c2* Mutant Can Be Rescued by the Expression of the Phospho-Dead EXO70C2 Variant!

Next, we tested the capacity of EXO70C2-PD and EXO70C2-PM variants fused to GFP and expressed under the control of the native *EXO70C2* promoter to compensate for the pollen tube growth defect seen in the Arabidopsis *exo70C2* mutant. We transformed homozygous *exo70C2* mutant plants with the two constructs, selected plants heterozygous for the pEXO70C2:EXO70C2:GFP -PD and -PM variants and performed the *in vitro* pollen growth assay. We separately measured lengths of both fluorescent (containing EXO70C2:GFP -PD or -PM variant in *exo70C2* background) and non-fluorescent (*exo70C2* only) pollen tubes within each samples and we compared them to the pEXO70C2:EXO70C2-WT:GFP lines, that had previously been shown to complement the *exo70C2* phenotype (Synek et al., 2017). Invariably, the length distributions of non-fluorescent pollen tubes from all the lines heterozygously expressing three EXO70C2:GFP variants in the *exo70C2* background were always significantly shorter than the wild type pollen tubes, confirming our previous observations (Figure 5B). The length distribution of the fluorescent EXO70C2-PD pollen tubes resembled that of the fluorescent EXO70C2-WT pollen tubes, thus showing the ability of the -PD variant to rescue the *exo70C2* phenotype (Figure 5B). On the other hand, the lengths of the fluorescent pollen tubes in the EXO70C2-PM-expressing plant were similar to or even shorter than that of *exo70C2* mutant pollen tubes. Collectively, these experiments confirmed the role of the phosphorylation sites in EXO7C2 in the context of pollen tube growth inhibition.

### AtEXO70C2 Phosphorylation May Affect Its Propensity to Induce Abnormal Pollen Tube Morphology

**FIGURE 6:**
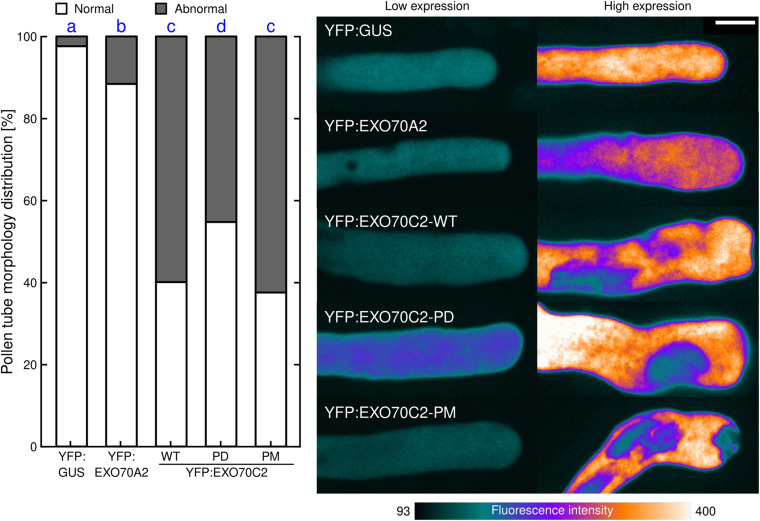
Changes in cell morphology induced by EXO70C2 are controlled by its phosphorylation status. Tobacco pollen tubes were transiently transformed with YFP:AtEXO70C2 wild type (WT), phospho-dead (PD), and phospho-mimetic (PM) variants and imaged 8 h after transformation by spinning disk confocal microscopy. Cells expressing YFP:GUS and Arabidopsis YFP:EXO70A2 were used for comparison. Left, frequencies of pollen tubes exhibiting normal or abnormal morphology (abberant cell shape, membrane invaginations). At least 61 cells per variant were imaged in three independent experiments. Different letters indicate significant differences between samples at *P* < 0.05. Right, typical images displayed using a color intensity code with the same pixel range set for all images (i.e., reflecting differences in protein expression levels). Bar 10 μm.

While analyzing the role of AtEXO70C2 phosphorylation in growing tobacco pollen tubes, we noticed that overexpression of the three *EXO70C2* variants caused substantial changes in the cell morphology, namely the formation of aberrant structures and shapes at the pollen tube tip. Conversely, such aberrations were rarely present when we transformed pollen tubes with YFP:GUS control and also with the canonical exocyst EXO70 subunit YFP:AtEXO70A2. To analyze this behavior quantitatively, we ranked the observed shapes and structures of pollen tubes first into two separate groups with either normal or abnormal pollen tube shape characterized by the formation of diverse apical invaginations or truncated pollen tube tip-morphology (Figure 6).

In control tobacco pollen tubes expressing YFP:GUS, we observed almost exclusively normal pollen tube tip expansion (98%). Similarly in EXO70A2-expressing pollen tubes, we observed largely normal growth (89%) and only exceptionally noticed aberrations similar to those observed in *EXO70C2*-expressing cells. When we compared the effects of the expression of the three EXO70C2 variants, we found that the expression of EXO70C2-PD produced less severe abnormal phenotype (Figure 6), as the normal distribution in this population was prevalent (55%) compared with the expression of the EXO70C2-WT or the EXO70C2-PM variant (40 and 38%, respectively). This phenotype is indicative of disturbed membrane trafficking and unbalanced exocytosis dynamics.

### EXO70C2 Phosphorylation-Mimicking Does Not Affect the Interaction With ROH1 in the Yeast Two-Hybrid Assay

**FIGURE 7:**
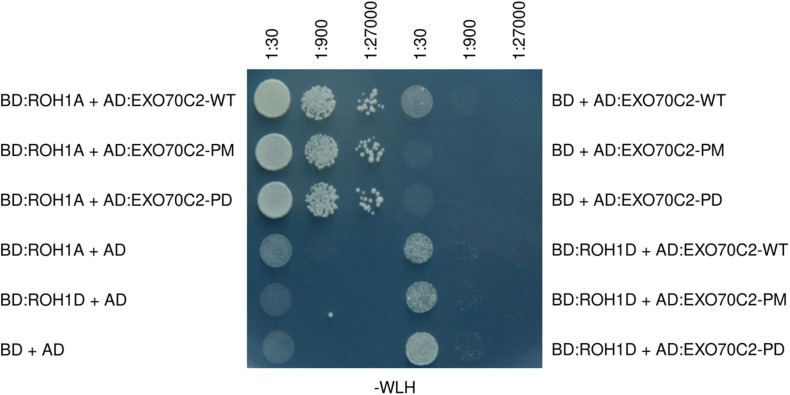
EXO70C2 shows different interaction capacity to distinct ROH1 paralogs, that is independent on its phosphorylation status. Yeast two-hybrid assay of two different members of the Arabidopsis ROH1 family (ROH1A and ROH1D) and the EXO70C2 in WT, phospho-mimetic (PM), and phospho-dead (PD) variants. ROH1 paralogs were fused with the DNA-binding domain (BD) of the yeast GAL4 transcription factor and EXO70C2 forms were fused with the activation domain (AD) of the transcription factor. BD or AD alone stand for the empty vectors as controls. Yeast strain AH109 was grown for 3 days on -TRP -LEU -HIS (-WLH) plates at 28°C.

AtEXO70C2 had previously been shown to interact with ROH1 in the yeast two-hybrid system. We therefore employed the yeast two-hybrid system to test the influence of the AtEXO70C2 phosphorylation sites on the interaction with ROH1 proteins. ROH1 proteins are EXO70 interactors and presumed negative secretion regulators (Kulich et al., 2010; Synek et al., 2017). We used already published interaction of AtROH1A and AtEXO70C2 as a positive control, and empty vectors as a negative control. Three different dilutions were used to eliminate possible dosage effects and none of the constructs conferred auto-activation. While clear interaction was observed between EXO70C2 and both ROH1 proteins, the strength of the interaction did not change for any of the three EXO70C2 variants (Figure 7 and Supplementary Figure S2). Interestingly, much weaker interaction was observed in case of ROH1D, a paralog of ROH1A, although the strength of the interaction was also not dependent on the putative phosphorylation status of AtEXO70C2 (Figure 7). We thus concluded that the phosphosite mutations do not affect the interaction with its ROH1 protein interactors.

## Discussion

Transcriptomic and proteomic data (Grobei et al., 2009; Loraine et al., 2013; Synek et al., 2017) drew our attention to the differential biological and biochemical roles of EXO70 isoforms in different plant tissues. In most, if not all, tissues several EXO70 subunits are expressed in the same cell (Zárský et al., 2009, 2013; Pečenková et al., 2011; Sekereš et al., 2017; Kulich et al., 2018). These data also indicate that different *EXO70* isoforms are co-expressed throughout pollen development. In Sekereš et al. (2017) it was already shown that most pollen-expressed *EXO70* genes were detected as orthologs both in *Arabidopsis* and several *Solanaceae* species including tobacco. In the case of *Arabidopsis thaliana EXO70C2* and *Nicotiana tabacum EXO70C2* isoforms orthology relationship was also proven based on a phylogenetic analysis (Sekereš et al., 2017). *EXO70C1*, *EXO70C2* and *EXO70H3* are the most highly expressed isoforms in both Arabidopsis and tobacco pollen (Sekereš et al., 2017). Also real-time PCR data for *EXO70C2* in Arabidopsis and tobacco showed high expression of this subunit at later stages of pollen development (Lai, 2016; Sekereš et al., 2017) indicating its important function in germinating and growing pollen tubes. Genetic analysis in Arabidopsis uncovered *EXO70C2* as a negative regulator of pollen tube polarized expansion (Synek et al., 2017). As LOF mutants are not available for tobacco, we used a protocol for AODN-imposed mRNA suppression using *in vitro* germinated pollen to test the function of tobacco *EXO70C2*. In the past, significant results have already been achieved not only in pollen to study polygalacturonase in tomato fruits (Smith et al., 1988) and chalcone synthase in petunia and tobacco plants (Krol et al., 1988). In the last decade, several studies based on the AODN strategy have been published in a variety of plant species (Sun et al., 2005; Liao et al., 2013).

As expected, based on the data from Arabidopsis, also tobacco pollen tube cultures with suppressed *EXO70C2* grew faster than wild type pollen – but did not burst in contrast to LOF mutant pollen as observed in Arabidopsis (Synek et al., 2017). This can be explained by only partial suppression of *EXO70C2* gene expression by AODNs. Next, we tested and compared *Arabidopsis thaliana* EXO70C2 YFP N- or C- terminal fusions in transient biolistic transformation assays in tobacco pollen tubes *in vitro*. We clearly demonstrated the dose-depend inhibitory effect of *EXO70C2* gene expression on pollen tube tip growth. We inferred that the N-terminal YFP-fusion is a stronger inhibitor than EXO70C2 C-terminal YFP fusion. This possibly indicates that EXO70C2 tagged at the C-terminus is functional, but C-terminal YFP probably interferes with the normal physiological protein activity and also protein stability. C-termini of EXO70s are known to be critical for direct membrane lipid interactions (Zárský et al., 2009; Pleskot et al., 2015; Sekereš et al., 2015; Mei and Guo, 2018). Defects in cell wall deposition in seed coats, xylem development, cell plates and trichomes have been well described for several Arabidopsis LOF exocyst mutants (Fendrych et al., 2010; Kulich et al., 2010, 2015; Li et al., 2013; Vukašinović et al., 2016). However, it was noted that pollen tubes of *exo70C2* Arabidopsis LOF mutants grow in erratic fashion and often faster than the wild type (Synek et al., 2017), indicating that EXO70C2 functions as a negative regulator or moderator of rapid cell expansion. In agreement with these observations, the transient expression of ectopic Arabidopsis EXO70C2 protein caused dramatic suppression of growth rate of tobacco pollen tubes when compared to the YFP:GUS and EXO70A2 controls. The wild type and the phospho-mimetic EXO70C2 variants also showed more pronounced widening and inhibition of pollen tubes in contrast to phospho-dead and two other control constructs. Similarly to tobacco pollen, mild overexpression of *EXO70C2-WT* in Arabidopsis led to the strong inhibition of pollen tube growth, which was significantly alleviated in *EXO70C2-PD*-expressing cells. On the other hand, only EXO70C2-WT and -PD variants, but not the -PM variant were able to complement the short pollen tube length phenotype of *exo70C2* mutant. The different outcomes of expression of *EXO70C2-PD* and *-PM* variants seen in wild type and *exo70C2* mutant cells suggests that the phosphorylation of EXO70C2 is more complex than a simple on-off switch. Differently phosphorylated and non-phosphorylated EXO70C2 isoforms may compete for the common interacting partners or regulate the biological activity of the other variant via negative feedback loops. EXO70C2 might negatively affect cell expansion by interfering directly with the exocyst or other putative exocyst regulatory or interaction molecules as for instance kinases or small GTPases.

Although the EXO70C2 exocyst subunit plays an important role in pollen tube growth regulation, no interaction with other exocyst subunits was detected so far. In turn, ROH1A protein was identified as an EXO70C2 interacting partner (Kulich et al., 2010; Synek et al., 2017). ROH1 proteins constitute a small plant-specific protein family that is related to BYPASS1 proteins, and both families form two phylogenetically distinct clades (Kulich et al., 2010). Gain-of-function (GOF) of the *ROH1A* gene in Arabidopsis plants resulted in of phenotypic deviations typical for LOF exocyst mutants (i.e., ROH1 GOF and exocyst LOF mutants show reduced pectinaceous seed coat development), which prompted us to propose a negative regulatory function for ROH1 in secretion (Kulich et al., 2010). It is therefore relevant that this protein does interact with EXO70C2, another negative regulator of the secretory pathway and cell wall biogenesis. We chose two pollen-expressed ROH1 paralogs, ROH1A and ROH1D, to test the importance of phosphorylation status of AtEXO70C2 for this direct interaction. Although our results have not indicated any changes in the interaction strength based on the mutations changing the phosphorylation status of EXO70C2 in the yeast two-hybrid assay, we observed an obvious preference, at least in this assay, of EXO70C2 for one of the two paralogs – ROH1A. We might speculate that this specialization of interactions is the reason why four of six *ROH1* paralogs are highly expressed in pollen (Genevestigator data). Interaction of EXO70C2 with ROH1 proteins might indicate a function of a hypothetical negative regulatory module of secretion.

Arabidopsis EXO70A2, another EXO70 subunit highly expressed in pollen, was used as an alternative control (Marković et al., 2020). We found that it also partly inhibited the speed of elongation and expansion but significantly less than EXO70C2 (Figure 4). Unlike EXO70C2, EXO70A2 is normally part of the assembled exocyst complex and we believe that ectopically expressed GFP:EXO70A2 competes with the endogenous tobacco EXO70A2 for the interacting subunits which ultimately leads to partial growth inhibition. Proteomic analyses of EXO70 family members in tobacco pollen in three different stages of pollen germination determined the presence of EXO70A2 and all EXO70 C-class members (Sekereš et al., 2017). Abnormal swelling of the tip-shape morphology was observed only exceptionally in YFP:EXO70A2-expressing pollen tubes. Our data for Arabidopsis EXO70A2 (Marković et al., 2020) show that this isoform is evolutionarily adapted to positively support pollen development, germination and pollen tube growth. Its interference with pollen tube growth of tobacco might be a result of the competition of endogenous tobacco EXO70A2 with the ectopically overexpressed Arabidopsis EXO70A2 for functional exocyst assembly. An important distinction is also that, while AtEXO70A2 is localized partially also to the plasma membrane at the very tip of the growing pollen tube, AtEXO70C2 was never recruited to the plasma membrane even after increasing the plasmid concentration, which confirms the notion that C-class EXO70 isoforms do not bind the plasma membrane as already showed in Sekereš et al. (2017) and Synek et al. (2017). This is also consistent with the observation that EXO70Cs do not interact with the core exocyst complex (Synek et al., 2017).

Apart from yet unidentified kinase(s) phosphorylating EXO70C2 and ROH1 proteins, possibly a number of other regulatory proteins are responsible for the regulatory activity of EXO70C2 through a direct or indirect action in polarity growth and localized cell wall biogenesis moderation. Certainly, there will be phosphatases able to dephosphorylate EXO70C proteins.

Using publicly available data from Arabidopsis SAM translatome (Tian et al., 2019) we spotted that on the background of almost zero expression throughout the SAM, EXO70C2 is the most significantly upregulated at the active border domain between the meristem and leaves/cotyledons (Supplementary Figure S3; Hibara et al., 2006). It is therefore possible, that EXO70C2 isoforms contributes to the active suppression or localization of cell expansion in angiosperms and that negative or moderating regulators of secretion and cell wall deposition regulates the fine-tuning of organ growth and development in plants. Our data show that phosphorylation plays an important role in the regulation of EXO70C2-mediated growth control. How the inhibitory action of EXO70Cs on cell expansion is excluded from the very expanding tip in root hairs and pollen tubes? It does not seem to be based on the exclusion of EXO70Cs from the tip cytoplasm, as both in pollen tubes and root hairs we observed EXO70C-GFP signal in whole expanding cell including the very tip. An important goal for the future will be also the identification of the kinases and phosphatases involved in EXO70C2 phosphoregulation.

## Data Availability Statement

The datasets presented in this article are not readily available because Restrictions apply. Requests to access the datasets should be directed to VŽ, zarsky@ueb.cas.cz.

## Author Contributions

AS, MP, PP, and MH performed the experiments. HS and CS contributed the material. VŽ and CS initiated the study. MP and VŽ planned and designed the experiments. AS, MP, CS, and VŽ wrote the manuscript with the input from MH, HS, and PP. All the authors read and approved the submitted manuscript.

## Conflict of Interest

The authors declare that the research was conducted in the absence of any commercial or financial relationships that could be construed as a potential conflict of interest.

## Footnotes

1 http://199.184.19.19/cgi-bin/soligo.pl

2 http://blast.ncbi.nlm.nih.gov/

3 https://ggplot2-book.org

## Supplementary Material

Supplementary Figure 1 Multiple protein alignment of angiosperm EXO70C family and all EXO70 paralogs from Arabidopsis.

Supplementary Figure 2 Yeast two-hybrid interaction assay for EXO70C2 and ROH1 members. Interaction assay of two different members of the Arabidopsis ROH1 family (ROH1A and ROH1D) and the Arabidopsis exocyst EXO70C2 subunit in its WT, phospho-mimetic (PM) and phospho-dead (PD) variants performed under stringent conditions **(A)**. Corresponding transformation controls are shown in **(B)**.

Supplementary Figure 3 EXO70C2 is specifically expressed in domain surrounding shoot apical meristem.
