# Supplementary figures and images for: Regulation of Exocyst Function in Pollen Tube Growth by Phosphorylation of Exocyst Subunit EXO70C2
Source: Front Plant Sci. 2021 Jan 14;11:609600. doi: 10.3389/fpls.2020.609600 (PMC7840542; doi:10.3389/fpls.2020.609600)

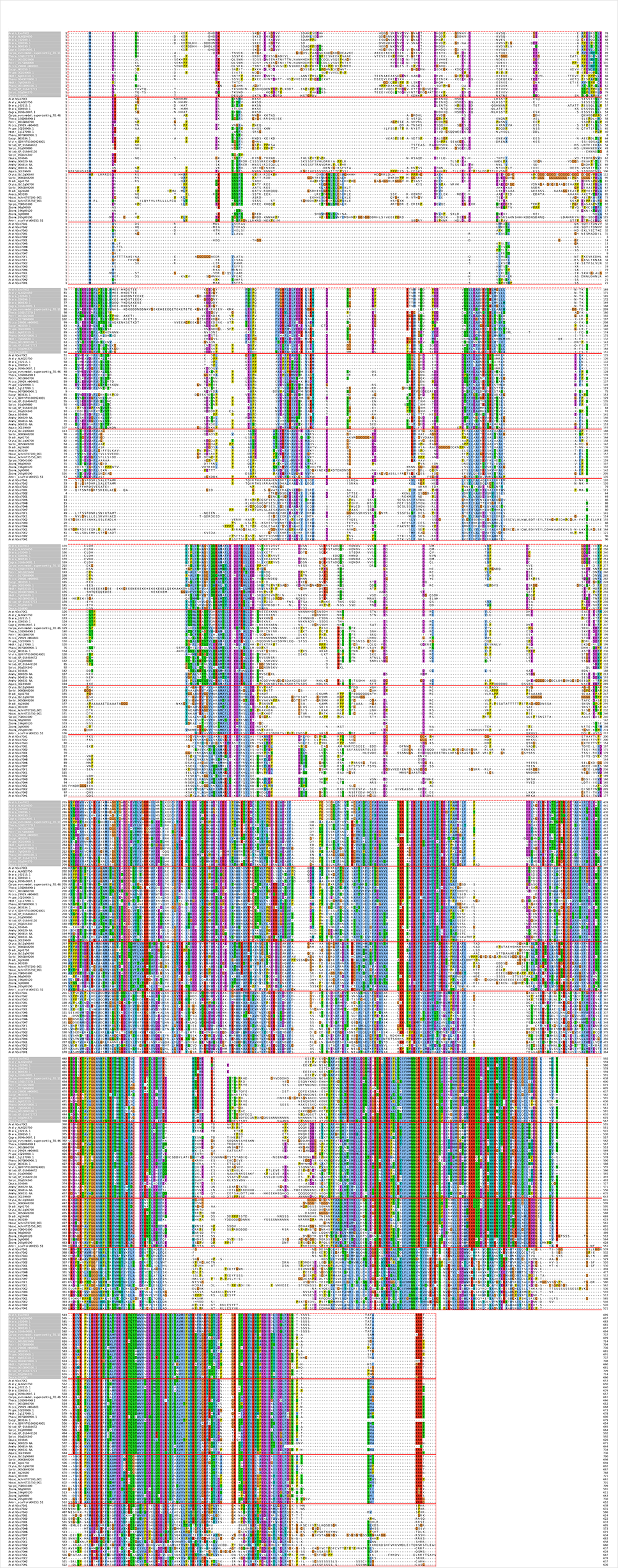

Supplement: Supplementary Figure 1 — Multiple protein alignment of angiosperm EXO70C family and all EXO70 paralogs from Arabidopsis. [file Image_1.TIFF]

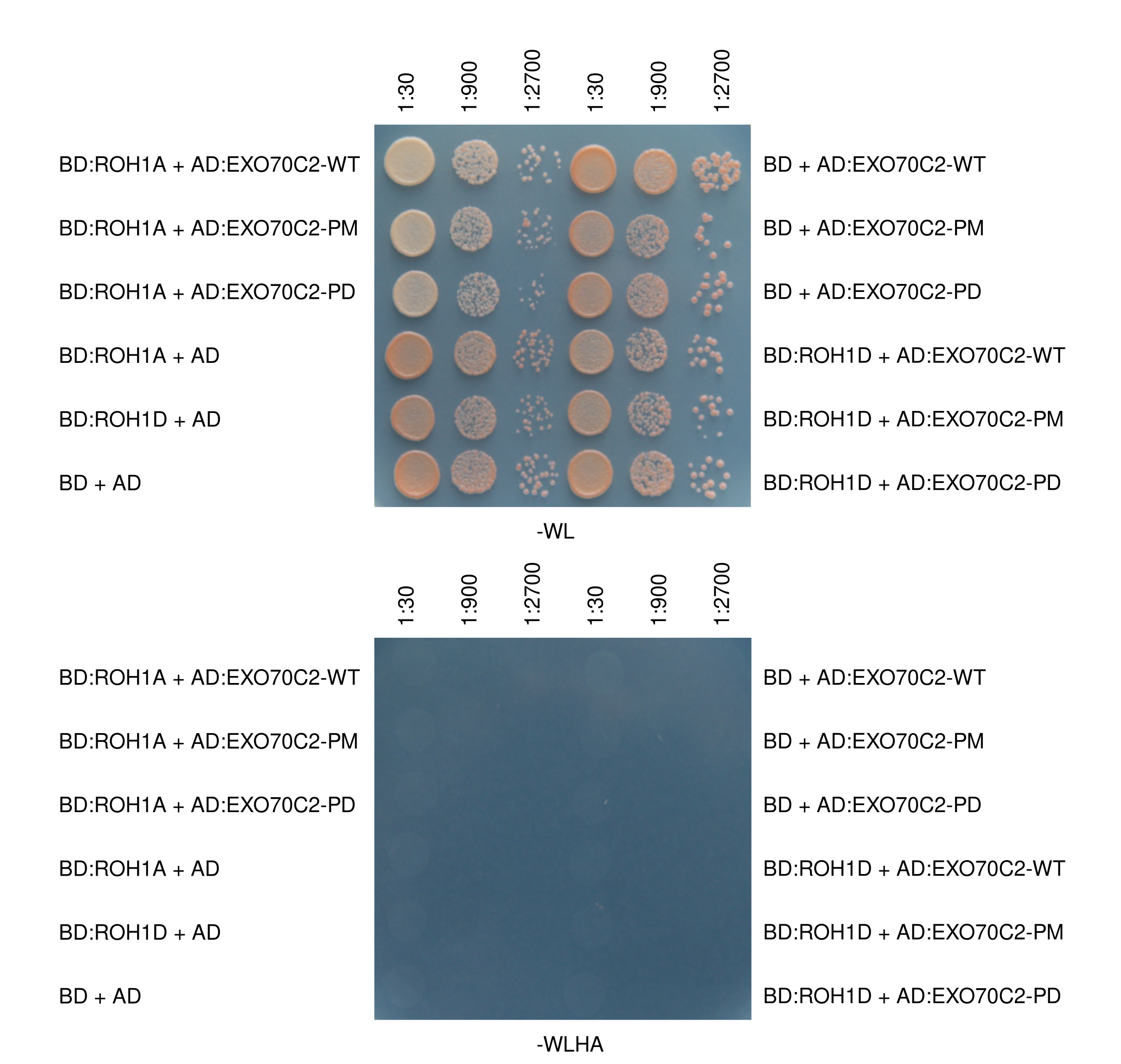

Supplement: Supplementary Figure 2 — Yeast two-hybrid interaction assay for EXO70C2 and ROH1 members. Interaction assay of two different members of the Arabidopsis ROH1 family (ROH1A and ROH1D) and the Arabidopsis exocyst EXO70C2 subunit in its WT, phospho-mimetic (PM) and phospho-dead (PD) variants performed under stringent conditions (A). Corresponding transformation controls are shown in (B). [file Image_2.TIFF]

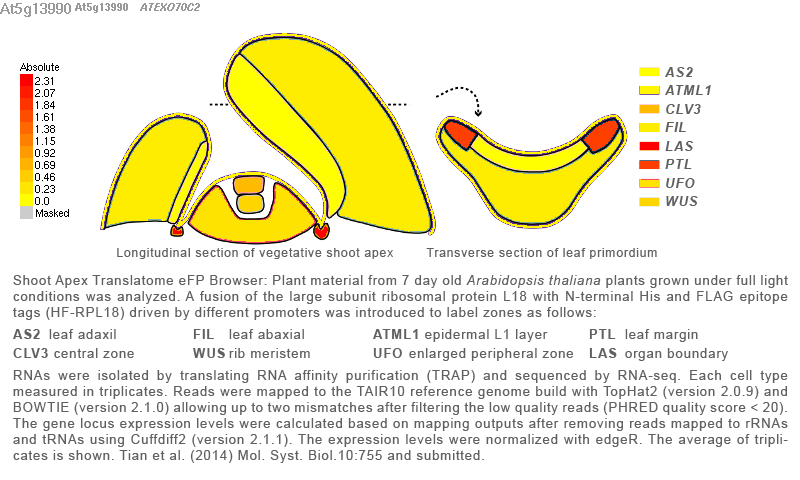

Supplement: Supplementary Figure 3 — EXO70C2 is specifically expressed in domain surrounding shoot apical meristem. [file Image_3.TIF]
